# Comparison between effects of different doses of *Melissa officinalis* and atorvastatin on the activity of liver enzymes in hypercholesterolemia rats

**Published:** 2014

**Authors:** Ali Zarei, Saeed Changizi Ashtiyani, Soheila Taheri, Fateme Rasekh

**Affiliations:** 1 *Department of Biology, Unit of Science Research, Islamic Azad University of Damghan, Damghan, I.R. Iran*; 2 *Department of Physiology, Arak University of Medical Sciences, Arak, I. R. Iran*; 3 ***Deputy of Research****, Arak University of Medical Sciences, Arak, I. R. Iran*; 4 *Department of Plant Biology, Payame-Noor University, Tehran, I. R. Iran*

**Keywords:** *Atorvastatin*, *Hypercholesterolemia*, *Melissa officinalis*, *liver enzyme*, *Rat*

## Abstract

**Objectives:** Liver is one of the most sensitive tissues to oxidant damage. Hence, the present study was conducted t**o **compare the effects of* Melissa officinalis *(MO) extract and Atorvastatin on the activity of **liver enzymes **in rats.

**Materials and Methods:** In this experimental study, 60 male Wistar rats were selected and allocated to six groups (n=10). The control group received a normal diet, sham group received a fatty diet while other groups received a fatty diet and the alcoholic extract of MO, at minimum (25 mg/kg), moderate (50 mg/kg), and maximum (75 mg/kg) doses (i.p.). The last group received Atorvastatin (10 mg/kg) through gavage with a fatty diet over a 21-day period. At the end of this 21-day period, blood samples were drawn and the levels of alanine aminotransferase (ALT), aspartate aminotransferase (AST), and alkaline phosphatase (ALP), activity of liver enzymes as well as cholesterol in the samples were measured.

**Results:** The obtained results showed that the activity of liver enzymes in the treatment groups receiving MO extract and the group receiving Atorvastatin decreased significantly. MO extract reduced the level of liver enzymes.

**Conclusion:** Therefore, further studies for obtaining a better understanding of the mechanism of effect of MO for treating liver diseases are recommended.

## Introduction

The study of compounds with herbal origin is an interesting branch of medicine (Akhondzadeh et al., 2003). Currently, a great deal of attention is paid to the consumption of fruits and vegetables for their protective effects against cancer, cardiovascular diseases, and liver diseases (Perez-Carreon et al., 2002; Pyo et al., 2004 ▶). 

This is due to the presence of antioxidant compounds including vitamin C, Vitamin B, **Carotenoids, **lycopens, and flavonoids that prevent damage caused by free radicals (Mitra et al., 1998; Sun, 2002; Yang et al., 2001). *Melissa officinalis (M. officinalis) *or Lemon balm belonging to the Lamiaceae family is a medicinal plant native to East Mediterranean region and West Asia. In Iran, this plant is known as Badranjbooye and grows largely in Tehran, Golestan, Azarbayjan, Lorestan, and Kermanshah (Emamghorishi and Talebianpour, 2009 ▶; Zargari, 1991 ▶). Since ancient times, this herb has been used for its antispasmodic, sedative, hypnotic, nutritional, anti-flatulent, and perspiration properties (Emamghorishi and Talebianpour, 2009 ▶; Blumenthal et al., 2000 ▶).* M. officinalis *also enhances memory and relieves stress (Akhondzadeh et al., 2003; Schulz et al., 1998 ▶) and it is applied to the treatment of sore throat, herpes, and headache (Emamghorishi and Talebianpour, 2009 ▶; Vessal et al., 1996).

 In Iran, it is used for treating bad-temper, anxiety, and nervousness in young girls and women (Emamghorishi and Talebianpour, 2009 ▶; Zargari, 1991 ▶; Shafie-Zadeh, 2002 ▶; Imami et al., 2003 ▶). *M. officinalis *can be effective in the treatment of fever, indigestion, insomnia, and epilepsy as well (De Carvalho  et al., 2011). One of the main properties of M. officinalis is its antioxidant property (Pereira et al., 2009) which is due to the presence of special compounds in it. M. officinalis extract has polyphenolic compounds, such as quercetin, gallic acid, as well as flavonoids, aldehyde, and tannin compounds (Pereira et al., 2009). Due to its medicinal properties, *M. officinalis *has been used in other studies for examining its effects on Alzheimer, memory, learning, and depression. However, to date no study has been done to compare the effects of *M. officinalis *extract *and *Atorvastatin on the amount of activity of liver enzymes in hypercholesterolemic rats. Therefore, the present study was done to compare the effects *of M. officinalis extract and *Atorvastatin on the activity of liver enzymes in hypercholesterolemic rats. 

## Materials and Methods

This experimental study was done on 60 male Wistar rats supplied from the Animal Breeding Center of Shiraz University of Medical Sciences. The rats were kept in standard conditions of temperature and light *ad libitum*. Animal care and handling was performed according to the guidelines and ethical codes set by the Iranian Ministry of Health and Medical Education for lab animals. Before launching the project, all of the rats were weighed to make sure that they were within the same range of weight (170±5 g).

The rats were randomly assorted into 6 groups of 10 each and received treatment for 21 days in the following way:

1) Control group: The rats did not receive any vehicle or drugs and had a normal diet.

2) Injection sham group: Hypercholesterolemic rats (cholesterol 2% was added to their food to render them hypercholesterolemic) were daily administered 0.2 ml of normal saline (i.p.).

3) Treatment group 1: Hypercholesterolemic rats were administered 25 mg/kg (minimum dose) of the alcoholic extract of *M. officinalis *(i.p.).

4) Treatment group 2: Hypercholesterolemic rats were administered 50 mg/kg (moderate dose) of the alcoholic extract of *M. officinalis *(i.p.).

5) Treatment group 3: Hypercholesterolemic rats were administered 75 mg/kg (maximum dose) of the alcoholic extract of *M. officinalis *(i.p.).

6) Atorvastain group: In this group, 10 mg/kg of atorvastain in the form of oral emulsion was administered to hypercholesterolemic rats through gavage. 

The experiment was carried out over a 21-day period and the injections were done through gavage everyday at 9 a.m. Injections were made intraperitoneally using insulin syringe and Atorvastatin (10 mg/kg) (Shafa Pharmaceuticals Co., Iran) was administered in the form of an oral emulsion. 

After this period, mild anesthesia using ether was done for obtaining blood samples from the rats heart to examine activities (IU/L) of serum liver enzymes **of **alanine aminotransferase (ALT), **aspartate aminotransferase** (AST), and alkaline phosphatase (ALP), as well as the amount of cholesterol in the samples. After centrifuging the blood samples at 3500 rounds per minute, serum samples were isolated and transferred to the lab for measuring the intended factors**. **Alanine aminotransferase (ALT) and **aspartate aminotransferase** (AST) were determined colorimetrically according to the method of Reitman and Frankel (Retiman and Frankel, 1957 ▶). Alkaline phosphatase (ALP) was assayed according to the method described by Belfield and Goldberg (Belfield and Goldberg, 1971).

**Analysis of cholesterol in plasma**

Plasma cholesterol level was determined by enzymatic colorimetric methods, using commercial kits from Pars Azmoon (Tehran, Iran) (Bhandari et al., 1998 ▶). Briefly, blood samples were transferred directly into centrifuge tubes, allowed to clot at room temperature for 20 min, and centrifuged for 20 min at 2000 rpm. The supernatant was transferred into test tubes for lipid analysis.

**Extraction method**

**Figure 1:**
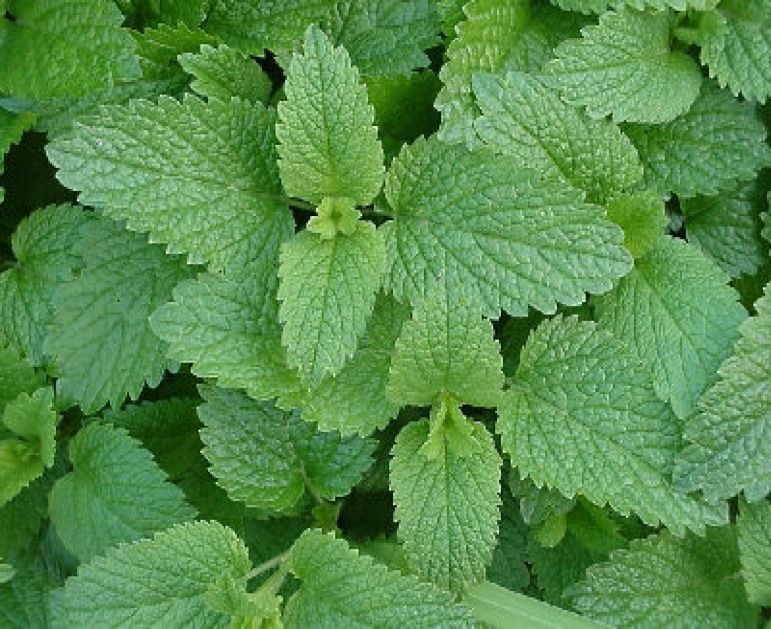
Melissa officinalis leaves

For preparation of the alcoholic extract of *M. officinalis *(Figure 1) standard methods of extraction were utilized. After providing *M. officinalis *plant, it was cleaned, dried, powdered, and eventually poured into a capped glass container and was mixed with ethanol alcohol 96%. The mixture was allowed to mix well for 72 hours. After that, it was first filtered and then centrifuged. The resulting mixture was placed in a hot bath to allow its alcohol evaporate completely. After this stage, due to the presence of water, the extract was still in a liquid state and for complete evaporation of water, it was kept at 40 ^°^C and then placed adjacent to sodium chloride.

**Preparation of high cholesterol 2% food**

For preparing high cholesterol 2% food, 20 g of pure cholesterol powder (Fluka Chemika) was solved in 5 ml of heated olive oil and was mixed with 1 kg of the rats food. All of the treatment groups received a fatty diet during the experiment. 

Data analysis

All obtained values are expressed as mean±Standard Error (SE) and data analyses were done using SPSS version 11.5 statistical software. The mean values were compared using one-way ANOVA analysis followed by **Tukey’s post-hoc** test. The level of significance was set at 0.05.

## Results

The comparison between the results of statistical analysis on the effects of *M. officinalis *extract and **Atorvastatin on the activity level of liver enzymes and **cholesterol in hypercholesterolemic rats (Table1) indicated that the amount of ALT and cholesterol in the injection sham group receiving a fatty diet significantly increased in comparison with the control group, whereas changes in all of the treatment groups receiving the extract and the group receiving Atorvastatin presented significant decreases compared with the injection sham group (p=0.00). However, none of the treatment groups presented significant changes compared with one another and the Atorvastatin group. 

In terms of AST, changes in the sham group were significantly higher than the control group, whereas its amounts in all of the treatment groups receiving* M. officinalis ***extract as well as** the Atorvastatin group had significantly decreased (p=0.001). 

ALP value in the sham group was significantly increased in comparison with the control group, whereas changes in all of the treatment groups receiving the extract and the group receiving Atorvastatin presented significant decreases compared with the sham group (p=0.00). Moreover, all treatment groups showed significant increases in ALP values compared with the Atorvastatin group (p=0.00). However, the treatment groups did not present significant changes compared with one another. Moreover, plasma concentrations of cholesterol in groups receiving different doses of the alcoholic extract of* M. officinalis ***showed significant reductions in comparison with the** injection sham **group **(Table 1). In this study, no significant differences in activity of liver enzymes between moderate dose of *M. officinalis *extract with its minimum and also maximum doses were found (Table 1). 

**Table 1:** The comparison between effects of different doses of Melissa officinalis (MO) versus Atorvastatin on activities (IU/L) of serum liver enzymes of alanine aminotransferase (ALT), aspartate aminotransferase (AST), alkaline phosphatase (ALP) and cholesterol in hypercholesterolemia rats

**Group**	**Control**	**Sham**	**MO** **Minimum dose (25 mg/kg)**	**MO** **Moderate dose (50 mg/kg)**	**MO** **Maximum dose (75 mg/kg)**	**Atorvastatin** **(10 mg/kg)**
**Parameters**						
**ALT (IU/L)**	55.87±3.7	80.37±6.7	58.75±2	62.96 ±2.2	68.12±1.4	65.28±2.4
**AST (IU/L)**	234.8±15.4	292±32.6	183±15.9	205±21.2	191±10.9	177.43±4.8
**ALP (IU/L)**	1017.3±71.8	2400±44.5	1485±63.9 †#	1520±95.9 †#	1454±10.5 †#	673.6±12.1
**Cholesterol** **(mg/dl)**	85±1	97.3±3.1	86.0±5.4	86±4.0	76.12±5.1	80.62±2

† Comparison with the sham group;

# Comparison* with the *Atorvastatin group*;*

* Comparison with the control group

## Discussion

Statistical analysis of the results showed that* M. officinalis *extract and Atorvastatin equally decreased liver enzymes. *M. officinalis *possesses strong antioxidant properties and it has phenolic compounds (Pereira et al., 2009). Polyphenolic compounds are among the most important anti-oxidants (Pérez-Carreón et al., 2002; Pyo et al., 2004 ▶; Toit et al., 2001 ▶). These compounds, especially flavonoids, have protective effects on liver damage induced by free radicals and liver toxins (Ahmad et al., 2002; Germano et al., 1999; Yoshikava et al., 2003 ▶).

*M. officinalis *is applied to the treatment of Alzheimer. Research has shown that this herb can calm the patients in their behavior, improve their learning, and enhance their short-term memory (Akhondzadeh et al., 2003). There was also considerable variation in cholinoreceptor interactions between different accessions of a M. officinalis species. M. officinalis extracts showed some nicotinic and muscarinic activity (Akhondzadeh et al., 2003; Wake et al., 2000 ▶). Moreover, it has also been shown that consumption of M. officinalis can be uplifting and brings about euphoria (Akhondzadeh et al., 2003; Kennedy et al., 2002). The findings of the present study indicated that similar to Atorvastatin, M. officinalis extract had an attenuating effect on activity of liver enzymes in the treatment groups. Polyphenolic compounds due to their antioxidant properties can neutralize free radicals in the environment and prevent their destructive effects (Perez-Carreon et al., 2002; Sun, 2000; Schulz et al., 2004 ▶).

Active free radicals, such as superoxide anions and hydroxyls, are capable of removing hydrogen atoms from the peripheral chains of saturated fatty acids in biologic membranes and result in lipid peroxidation **(**Kelly and Husband, 2003 ▶; Nazari et al., 2005 ▶**).** Since *M. officinalis *has quercine compounds which can inhibit lipid peroxidation (Pereira et al., 2009; -Saija et al. 1995), its extract stabilizes cell membrane and prevents the oxidation of membrane lipids. Hence, in the present study, decrease in activity of **liver enzymes **in the treatment groups receiving *M. officinalis *is justifiable. Moreover, the presence of non-enzymatic antioxidants, including vitamin E, beta carotene, and vitamin C in this herb justifies its ability to inhibit damage due to free radicals. The involved enzymes are superoxide dismutase, catalase, and glutathione peroxidase. When the anti-oxidant defense system is lost in the body, increase in the formation of free radicals can cause cellular oxidative stress. Lipid peroxidation and oxidative stress severely increase in patients with diabetes and hyperlipidemia (Kelly and Husband, 2003 ▶; Nazari et al., 2005). 

Polyphenolic compounds and flavonoids can also revive the cells against glutathione depletion and protect them by increasing the capacity of anti-oxidant enzymes (glutathione, glutathione reductase, glutathione peroxidase, and catalase) (Sanz et al., 1998; Al–Qarawi et al., 2002).

Free radicals damage cell membrane, such as in hepatocytes. With destruction of hepatocytes membrane, the activity of liver enzymes increases which results in the expression of these enzymes, which are normally in cytosol of cells. Increase in the activity of these enzymes explains the type and degree of liver damage (Shariati and Zarei, 2006 ▶). Considering the properties of this herb which decrease the production of free radicals and its anti-oxidant and anti-inflammatory properties, reduction in the level of activity of liver enzymes in the treatment groups receiving* M. officinalis *extract is predictable. In the present study, it was shown that treatment with *M. officinalis *extract can protect liver cells which are probably due to the presence of flavonoids.

Research has shown that* M. officinalis *possesses anti-inflammatory properties. There is a direct relationship between the amount of lipids and leptin. Furthermore, with increase in the amount of lipids and their deposition in the liver, the level of activity of **liver enzymes **increases. Increase in the amount of lipids results in an increase in the level of leptin (Saeb et al., 2010 ▶; Jelodar and Nazifi habibabad, 1997 ▶, Bush, 1992 ▶; Tohidi, 2007 ▶). Leptin secretion increases with stimulation of inflammation and it increases hormonal and cellular immune responses (Gainsford et al., 1996). Moreover, adipocytes release various protein signals that include several cytokines such as TNF-α, IL-6, and absorbing proteins (Hersoug and Linneberg, 2007). By activating the secretion of TNF-α and IL-6 from **mononucleus** cells in cytokines, leptin can cause inflammation (Gatreh-Samani et al., 2011 ▶). TNF-α increase is probably followed by liver necrosis which leads to an increase in activity of liver enzymes (Shariati and Zarei, 2006 ▶). 

Noticing the anti-oxidant and anti-inflammatory properties of *Melissa officinalis,* reduction in the level of activity of **liver enzymes **in the treatment groups was predictable. Alkaline phosphatase is a transpeptidase which increases in liver and bone diseases. Research has shown that the phenolic compound present in medicinal plants can prevent the toxic effects of drugs on liver and cause the release of serum **ALT** into blood. Studies have demonstrated that hyperlipidemia levels, glycerides, and cholesterol in groups under treatment with fatty diets increase (Pourjafar et al., 2010 ▶).

Atorvastatin is one of the chemical drugs that are used for treating hyperlipidemia. Drugs reducing blood fat, such as lovastatin and Atorvastatin, by inhibiting the channel of hydroxymethylglutaryl-coenzyme, a reductase synthesis, and decreasing the secretion of VLDL containing triacylglycerol and bad cholesterol by liver can decrease plasma cholesterol.

The results of a study by Lee and coworkers showed that *in vivo* and *in vitro* treatments of Ob-X (a mixture of three herbs, *Morus alba*, *Melissa officinalis*, and *Artemisia capillaries*) regulate serum lipid profiles, adipose tissue mass, and body weight gain by inducing the mRNA expression of peroxisome proliferator-activated receptor (PPAR) target genes responsible for fatty acid oxidation in high-fat diet-fed obese mice. Thus, hepatic PPAR seems to be a crucial component for Ob-X regulated energy metabolism and Ob-X may be very useful for treating obese patients with hypercholesterolemia and hypertriglyceridemia.

In general, methods of reducing cholesterol include decreasing its excretion and inhibiting its synthesis and absorption. Studies done on *M. officinalis *also indicate that it contains phenolic alkaloids that are among the materials which can inhibit cholesterol synthesis (Pereira et al., 2009; Ashtiyani et al., 2011 ▶). The results of this study is in agreement with Bolkent S and coworkers that showed that the administration of M. officinalis L. extract reduced total cholesterol, total lipid, ALT, AST, and ALP levels in serum as well as lipid peroxidation (LPO) levels in liver tissue. Moreover, they showed increased glutathione levels in the tissue (Bolkent et al, 2005). As a result, it was suggested that *M. officinalis *extract exerted a hypolipidemic effect similar to **Atorvastatin** and showed a protective effect on the liver of hyperlipidemic rats. 

Previous studies and studies done together with this one show that *M. officinalis *extract by decreasing AST, ALP, and ALT as well as cholesterol is effective in improving the function of lever and treating liver diseases.
